# Clinical predictors of surgical outcomes of severe carpal tunnel syndrome patients: utility of palmar stimulation in a nerve conduction study

**DOI:** 10.1186/s12891-020-03750-z

**Published:** 2020-11-07

**Authors:** Yuichi Sasaki, Tohru Terao, Emiko Saito, Keiichiro Ohara, Shotaro Michishita, Naoki Kato, Satoshi Tani, Yuichi Murayama

**Affiliations:** 1 Department of Neurosurgery, Atsugi City Hospital, 1-16-36 Mizuhiki, Atsugi-shi, Kanagawa 243-8588 Japan; 2 Department of Neurosurgery, The Jikei University Hospital, Tokyo, Japan

**Keywords:** Carpal tunnel syndrome, Nerve conduction study, Bland’s classification, Axonal degeneration

## Abstract

**Background:**

Carpal tunnel syndrome is a common peripheral nerve compression disorder. However, there is no established opinion regarding the predictors of symptom improvement after surgery. This study aimed to identify the predictors of surgical outcomes of severe carpal tunnel syndrome patients.

**Methods:**

In the patients who underwent a carpal tunnel syndrome surgery, we selected the patients who had a preoperative Bland’s classification of grade 5 or 6, and assessed for the changes in Bland’s classification grade before and after surgery. Those who showed improvement from preoperative grades 5–6 to postoperative grades 1–4 comprised the improvement group. In contrast, those who did not show improvement and had postoperative grades 5 or 6 comprised the non-improvement group. In a nerve conduction study, amplitudes of the compound muscle action potential and sensory nerve action potential of the palms were assessed between the improvement and non-improvement groups.

**Results:**

Among the 60 hands of 46 patients who had a preoperative Bland’s classification of grade 5 or 6, 49 hands of 37 patients comprised the improvement group, and 11 hands of 9 patients comprised the non-improvement group. The amplitudes of the compound muscle action potential and sensory nerve action potential of the palms before surgery were significantly higher in the improvement group. The degree of improvement in Bland’s classification grade was correlated with the degree of clinical symptom improvement.

**Conclusions:**

Amplitudes of compound muscle action potential and sensory nerve action potential before surgery induced by palmar stimulation can predict improvements in nerve conduction study scores and clinical findings after surgical treatment.

## Background

Carpal tunnel syndrome (CTS) is the most common type of peripheral nerve compression disorder. It is known that surgical treatment for CTS is effective [1, 2], and some studies have attempted to identify predictors of surgical outcomes. As epidemiological and physical factors that predict postoperative symptom improvement, age, sex, and preoperative motor function were examined for the related functional outcomes after surgery [3, 4]. Socioeconomic factors in the workplace such as exposure to force and repetitive tasks, type of work, and degree of income may influence functional convalescence after surgery [5, 6]. The previous studies have shown that the clinical outcomes after surgery depend on multiple factors; there is no established opinion regarding predictors of symptom improvement after surgery.

This study aimed to identify the predictors of surgical outcomes in severe CTS patients. We performed a nerve conduction study (NCS) for the electrodiagnosis of CTS. In addition to general evaluation items, we measured the compound muscle action potential (CMAP) and sensory nerve action potential (SNAP) on palmar stimulation. By comparing the amplitudes of the CMAP and SNAP in the wrist and palm, we evaluated the degrees of conduction block and axonal degeneration at the pre and postoperative stages. These findings are important for the preoperative prediction of the degree of postoperative improvement in clinical symptoms of patients with severe CTS.

## Methods

### Measure of NCS and CTS diagnosis at our hospital

**Fig. 1:**
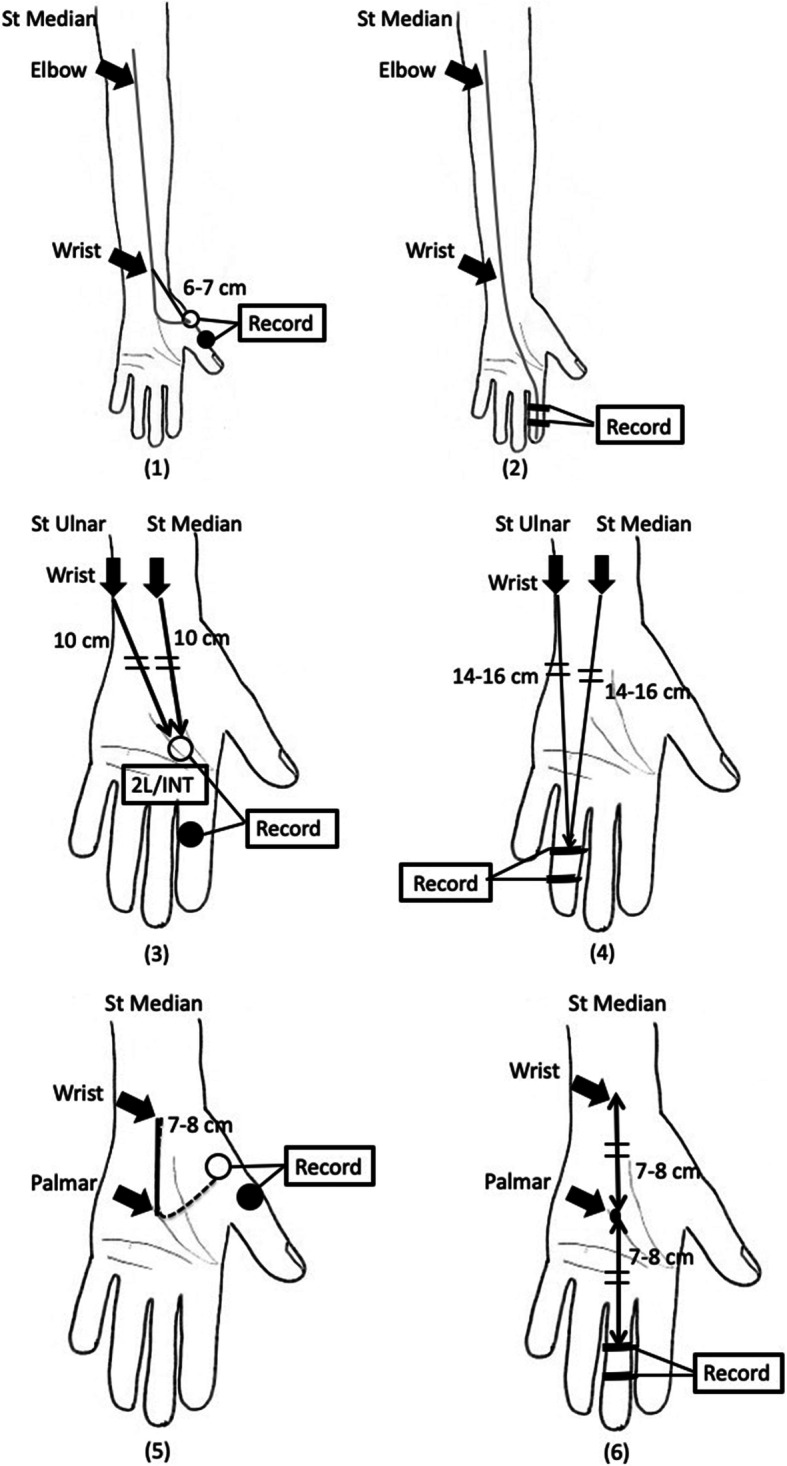
Nerve conduction study of carpal tunnel syndrome. (1) A motor nerve conduction study (MCS) was performed with a surface electrode on the abductor pollicis brevis (APB) and stimulation of the median nerve at the wrist and elbow. (2) A sensory nerve conduction study (SCS) was performed with a surface electrode on the second finger and stimulation of the median nerve at the wrist and elbow. (3) The compound muscle action potential (CMAP) was recorded with a surface electrode on the second lumbrical and the first palmar interosseous and stimulation of the median nerve and ulnar nerve. (4) The sensory nerve action potential (SNAP) was recorded with a surface electrode on the fourth finger and stimulation of the median nerve and ulnar nerve. (5) The CMAP was recorded with a surface electrode on the APB and stimulation of the median nerve at the wrist and the palmar region. (6) The SNAP was recorded with a surface electrode on the third finger and stimulation of the median nerve at the wrist and the palmar region. St Median: stimulation of the median nerve; St Ulnar: stimulation of the ulnar nerve

At our hospital, patients suspected to have CTS were initially assessed by neurologists for clinical histories; their physical conditions were examined. Physical examinations included assessing the responses to the Tinel and Phalen tests and the thenar eminence, specifically, observing thenar muscle wasting according to the American Academy of Orthopaedic Surgeons guidelines [7]. We performed an NCS for all patients diagnosed with CTS based on clinical symptoms and provocation test results [8, 9]. During the NCS procedure at our hospital (Fig. 1), CTS was diagnosed if the CMAP terminal latency (TL) indicated a delay of 4.0 ms or more during a motor NCS (surface electrode on the abductor pollicis brevis [APB] and stimulation of the median nerve at the wrist and elbow), the sensory nerve conduction velocity was less than 50 m/s during a sensory NCS (surface electrode on the second finger and stimulation of the median nerve at the wrist and elbow), and the TL of the median nerve was 0.4 ms or longer compared with that of the ulnar nerve during an assessment of the CMAP (surface electrode on the second lumbrical and the first palmar interosseous and stimulation of the median nerve and ulnar nerve) and SNAP (surface electrode on the fourth finger and stimulation of the median nerve and ulnar nerve). Furthermore, by stimulating the palmar region of the median nerve during assessment of the CMAP (surface electrode on the APB) and SNAP (surface electrode on the third finger), measuring the amplitudes of the CMAP and SNAP, and comparing the amplitudes of the wrist and palm, we can evaluate the motor and sensory nerve demyelination status (conduction block) and the number of remaining axons [10]. The same laboratory technician (E.S) performed the NCS and severity classification using the following Bland’s classification [11]; grade 0 represents no neurophysiology abnormality, grade 1 is a very mild abnormality (detected only in two sensitive tests), grade 2 is mild CTS (sensory conduction velocity from index finger to wrist < 40 m/s with motor terminal latency from wrist to ABP < 4.5 ms), grade 3 is moderately severe CTS (motor terminal latency > 4.5 ms and < 6.5 ms with preserved index finger SNAP), grade 4 is severe CTS (motor terminal latency > 4.5 ms and < 6.5 ms with absent SNAP), grade 5 is very severe CTS (motor terminal latency > 6.5 ms), and grade 6 is extremely severe CTS (surface motor potential from APB < 0.2 mV).

### Surgical criteria and surgical method for CTS at our hospital

We performed surgery for patients with impaired motor nerves classified as grade 3 or higher according to Bland’s classification and who do not show improvement with conservative treatment. In particular, for patients with cases classified as grade 5 or 6 and who showed severe impairment in the motor nerves that control the APB, we recommended surgery as the continuation of conservative treatment could cause APB atrophy.

The same surgeon (T.T) performed the surgery for all patients. All patients underwent surgery under a magnifying glass. After the upper arm was fastened with a tourniquet, surgery was initiated under local skin anesthesia with 1% epinephrine-containing lidocaine. A median longitudinal incision of approximately 35 mm that did not exceed the distal carpal line and a transverse incision of approximately 10 mm on the ulnar side of the proximal side were created. The palmar longus aponeurosis was split bilaterally to reveal the flexor ligament underneath. After checking the thickness of the flexor ligament and the presence or absence of calcification, the flexor ligament was carefully cut from the distal side to the proximal side without damaging the median nerve. In the proximal region, the flexor ligament may be thick and calcified; therefore, careful attention is necessary to ensure that the proximal operative field is fully deployed and that the proximal side of the flexor ligament remains uncut. The standard site for the distal incision is the perineural adipose tissue. After confirming sufficient decompression of the median nerve, compression from the tourniquet was released, the surrounding bleeding was stopped, and the wound was closed with a 4–0 nylon thread mattress suture [12].

### Materials and measurements

This study was approved by the local ethics committee of Atsugi City Hospital (approval number R1–06). Overall, 93 patients (129 hands, 74 right and 55 left hands) with CTS underwent carpal tunnel surgery at our hospital between April 2014 and March 2019. The mean age was 67.1 ± 12.4 years; 26 patients were males, and 67 were females. The 60 hands (37 right hands and 23 left hands) of 46 patients with a preoperative Bland’s classification of grade 5 or 6 were assessed for the changes in Bland’s classification grade before surgery and 6 months after surgery. The mean age of these 46 patients was 66.2 ± 12.9 years, and 13 were males, while 33 were females. Among patients with a preoperative Bland classification of grade 5 or 6, those who showed improvement to postoperative grades 1–4 were included in the improvement group, whereas those who did not show improvement and had postoperative grade 5 or 6 were included in the non-improvement group. Amplitudes of the CMAP and SNAP of the palms in preoperative NCS were compared in the two groups. The Wilcoxon rank-sum test was used for statistical analyses. Numerical values are shown as mean ± standard deviation. A *p*-value < 0.05 was considered statistically significant.

Furthermore, we assessed for the degree of numbness of the fingers, primarily in the thumb and index, middle, and ring fingers; the presence or absence of APB atrophy; and the presence or absence of opposition movements of the thumb before surgery. The patients were asked to complete a question postoperatively to assess changes in clinical symptoms [13]. Six months postoperatively, patients were asked to compare their symptoms with those before surgery, rate them, and select any of the following five options: cured, much better, better, unchanged, and worse. Based on these results, the correlation between changes in Bland’s classification and the degree of clinical symptom improvement before and after surgery was examined. Informed consent for the procedure was obtained from all patients, as was permission to use their anonymized data.

## Results

Numbness of the fingers improved in all patients, while the grade of the improvement varied depending on the preoperative Bland’s classification grade. There were no complications associated with the surgery in a preoperative Bland’s classification of grade 5 or 6.

**Fig. 2:**
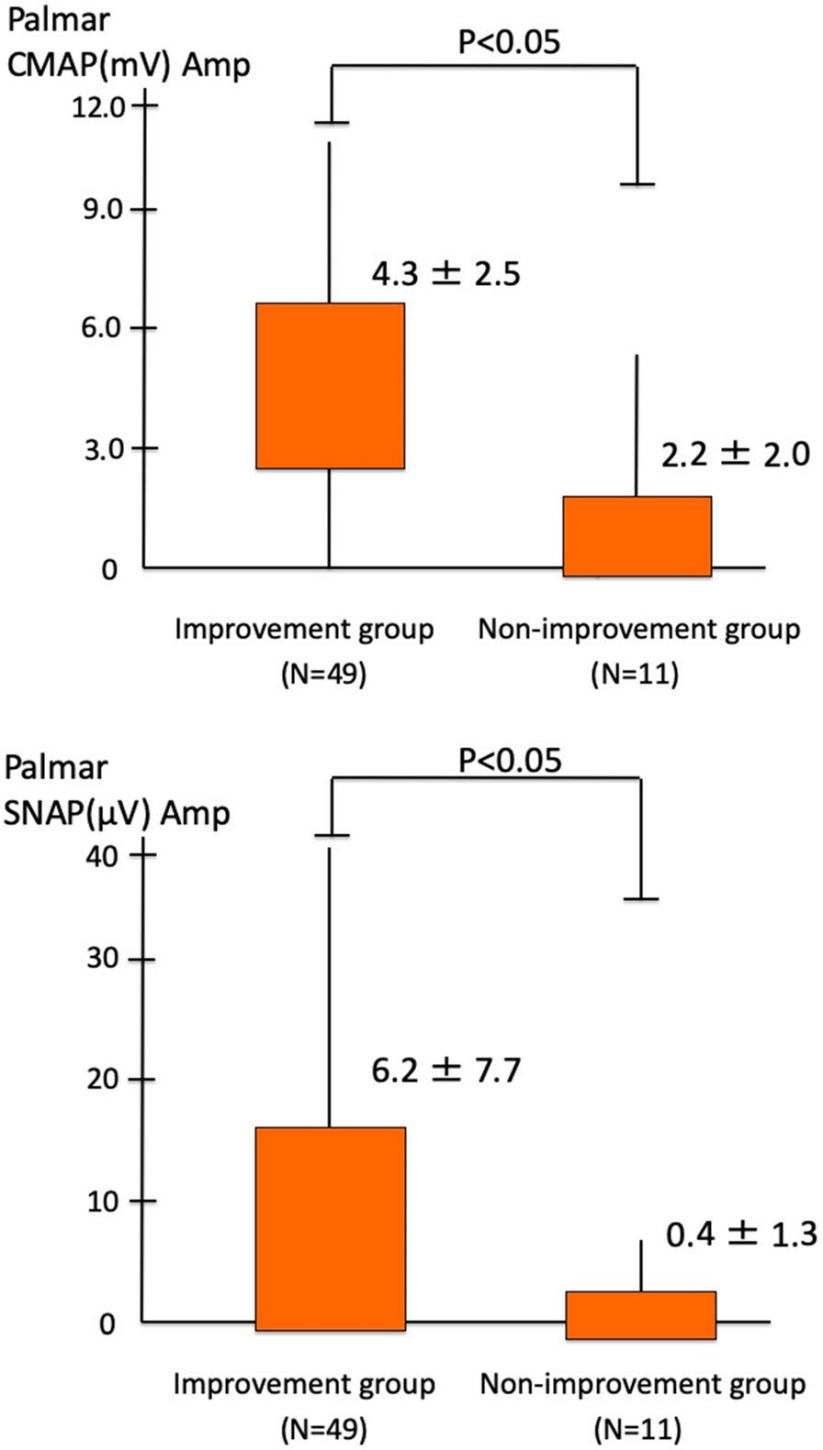
Comparisons of the compound muscle and sensory nerve action potential amplitudes between the two groups. These were recorded during stimulation of the palmar region before surgery. The palmar CMAP and SNAP amplitudes in the non-improvement group were severely reduced or disappeared when compared with the findings of the improvement group (*p* < 0.05, Wilcoxon rank-sum test)

Among the 60 hands of the 46 patients with a preoperative Bland’s classification of grade 5 or 6, 49 hands of 37 patients were included in the improvement group (Table 1). The mean age of the improvement group was 65.8 ± 13.2 years; 8 were males, and 29 were females. In contrast, 11 hands of 9 patients were included in the non-improvement group. The mean age of this group was 67.9 ± 11.6 years; 5 were males, and 4 were females. Of the 11 hands in the non-improvement group, 4 hands remained grade 6, 5 hands improved from grade 6 to grade 5, and 2 hands remained grade 5. In the 11 hands, the preoperative amplitudes of the CMAP of the palms were null in 3 hands, extremely low in 8 hands. The preoperative amplitudes of the SNAP of the palms were null in 10 hands, extremely low in 1 hand in the same group (Table 2). The amplitudes of the CMAP and SNAP of the palms before CTS surgery were significantly higher in the improvement group than in the non-improvement group (*p* < 0.05, Wilcoxon rank-sum test) (Fig. 2).

**Table 1:** Comparison of the preoperative and postoperative grading scale scores of the improvement group

Preoperative score	Postoperative score	N
5	1	1
	2	2
	3	30
	4	4
6	3	10
	4	2

**Table 2:** Comparison of the preoperative and postoperative grading scale scores of the non-improvement group

Patient age (y) and sex	Preoperative score	Preoperative wrist amp CMAP (mV)/SNAP (μV)	Preoperative palmar amp CMAP (mV)/SNAP (μV)	Postoperative score
74, M	5	0.38/null	0.36/null	5
81, M	6	null/null	null/null	5
81, M	6	null/null	null/4.5	5
62, F	6	null/null	1.8/null	6
61, F	6	0.09/null	2.8/null	5
91, F	6	null/null	null/null	6
68, M	6	null/null	3.6/null	6
55, M	6	1.7/null	2.2/null	5
55, M	6	3.3/null	5.1/null	5
59, M	5	1.2/null	2.3/null	5
60, F	6	null/null	6.0/null	6

*M* Male, *F* Female, *mV* Millivolts, *μV* Microvolts, *amp* Amplitude, *CMAP* Compound muscle action potential, *SNAP* Sensory nerve action potential

Table 3 shows the results of the question survey regarding postoperative clinical symptoms. In the improvement group, symptoms disappeared or markedly improved in 82% of cases, whereas symptoms improved to a small degree or very poor in the non-improvement group. The degree of improvement in Bland’s classification grade was correlated to the clinical symptom improvement. In the improvement group, 21 of the 49 hands (43%) had APB atrophy and thumb opposition movement disorder, whereas 7 of the 11 hands (64%) had APB atrophy and thumb opposition movement disorder in the non-improvement group. The clinical differences were independent (*p* = 0.182, chi-squared test).

**Table 3:** Results of the questionnaire with a 5-point rating scale after surgery

	Cured	Much better	Better	Unchanged	Worse
Improvement group (*n* = 49)	24 (49%)	16 (33%)	9 (18%)	0	0
Non-improvement group (*n* = 11)	0	2 (18%)	9 (82%)	0	0

## Discussion

Regarding the predictors of improvement in clinical symptoms following CTS surgery, Katz et al. [14] reported that multiple factors, including the history of tobacco use, history of alcohol consumption, old age, prolonged illness, male sex, and nocturnal symptoms, hinder symptom improvement. Unusual preoperative physical changes as the presence of atrophy of the thumb muscle [15], also complications of spring fingers, neuropathy of the ulnar nerve, metacarpal arthropathy of the thumb, and wrist arthropathy may be the factors which obstruct postoperative symptomatic improvement [16]. As mentioned, studies that have examined the predictors of postoperative symptom improvement have not demonstrated a specific perspective and lack objectivity. No studies have examined predictors of single-factor improvement in symptoms.

Garg et al. [17] examined the predictors of thumb opposition recovery after CTS surgery. In this study, patients suffering from severe CTS with thenar atrophy and detectable CMAP of the wrist showed promising improvements following CTS surgery. Furthermore, magnetic resonance neurography findings were also useful as predictors of thumb opposition recovery.

Regarding severe CTS with undetectable CMAP of the APB, Ebata et al. [18] reported variations in thenar muscle innervation. Patients with CTS who had undetectable CMAP of the APB were classified into four group, and over 10% of patients with severe CTS had mild or no muscle atrophy as well as intact thumb opposition, and the thenar muscle of their hands were innervated more by the ulnar nerve.

Regarding the evaluation and functional prognosis of axonal degeneration during an NCS, Caetano et al. [19] examined the severity of axonal degeneration by comparing the amplitude of the SNAP induced by wrist and palm stimulation. In this study, only the extent of axonal degeneration and functional prognosis of the SNAP were discussed. When the SNAP amplitude induced by palmar stimulation was 36.6 μV or less, these patients were defined as sever CTS without the CMAP of the palm into consideration because of the complexity of the measurement technology.

In our clinical study, we researched the correlation between the preoperative electrophysiological evaluation and postoperative symptom improvement. The results showed that the measurement of the amplitudes of the CMAP and SNAP from palmar stimulation, indicators of the number of remaining axons at the periphery of the palm, was useful for the evaluation of the preoperative predicted factors. The number of residual axons of the non-improvement group at the palm was lower than that of the improvement group, and an extreme decrease or disappearance of the preoperative SNAP and CMAP amplitudes in the non-improvement group was demonstrated on this fact. The APB atrophy appears as a result of the decrease in the number of axonal fibers at the palm, as shown in almost cases of the non-improvement group, and that contributed to the reason for the low degree of improvement in clinical symptoms even after surgical decompression.

By contrast, if the majority of remaining axonal fibers in the periphery from the palm that demonstrate no decrease in the amplitudes of the CMAP and SNAP were shown, the functional and organic recovery, as well as the improvement of Bland’s classification, would be observed after surgery by remyelination of motor and sensory nerve fibers and reinnervation. Therefore, evaluations of the amplitudes of the CMAP and SNAP in the palm are useful for creating an informed prediction before surgery, explaining the significance of the treatment, and improving postoperative clinical symptoms.

### Limitations

This study had a few limitations. It included a small number of patients who participated in a short follow-up. Therefore, further investigations involving a larger number of cases may be needed to determine more definitive conclusions.

## Conclusions

The findings of this study suggest that the amplitudes of CMAP and SNAP before surgery induced by palmar stimulation can be a predictor of improvement in NCS scores and clinical findings after surgical treatment.

## Data Availability

The datasets used and/or analyzed during the current study are available from the corresponding author on reasonable request.

## Abbreviations

APB: Abductor pollicis brevis
CMAP: Compound muscle action potential;
CTS: Carpal tunnel syndrome
NCS: Nerve conduction study
SNAP: Sensory nerve action potential
TL: Terminal latency
